# Treatment of Serous Effusions by Limitation of Fluid

**Published:** 1878-06

**Authors:** 


### TREATMENT OF SEROUS EFFUSIONS BY LIMITATION OF FLUID

Dr. Cheadle remarks that to pour fluid into the body, freely, at the same time that we are endeavoring to draw it out by all the means in our power seems illogical. The fluid drawn off from the blood by active measures will be replaced from the readiest source, the natural channel of supply, the absorbents of the alimentary canal, whose proper function it is to furnish it, if they have enough to draw upon. It is surely, he remarks, prima facie probable that if we lessen the means of supply through the gastro-intestinal vessels, the endosmotic drain upon the other means of supply, the effused fluid set up by the removal of fluid from the blood by purges and diuretics, will be intensified and prolonged, and thus rendered effectual when otherwise it would hardly have been called into action. What the blood fails to receive from the easier and natural source it will drink up from the abnormal source, and the equilibrium of the blood density be restored by the return into it of fluid previously forced out of it by the inflammatory process or by mechanical pressure, when mechanical pressure is lessened and density increased by the action of purges and diuretics. This theory seemed to him, in its general principle, so reasonable that he determined to put it in practice at the first convenient opportunity. He has been able to apply it in five cases; two of these were cases of cardiac dropsy, one of ascites, and two of ordinary pleuritic effusion.—*Lancet.*

---

The specialist in medicine is one who, limiting his attention to one or more detached portions of his art, specially neglects other parts which are essential to the making of a true physician.—*Barnes.*

He who attempts to do every thing does nothing thoroughly, or at least does many things imperfectly. The field of medicine has become so extended that no one can fully embrace the whole, indeed can cultivate properly, but a very small territory. The foundation principles of medical science every one engaged in any particular of the healing art, should understand and their practice confined to that in which the greatest efficiency can be attained.

Would Mr. Barnes apply to the general physician or to the dentist for treatment of diseased teeth? There is a considerable admixture of fallacy and nonsense in these thrusts at specialists.—[Ed Reg].

